# Dextran sulfate inhibits the invasion, migration, and programmed death-ligand 1 expression in human gastric cancer cells by affecting the M2 tumor-associated macrophage polarization

**DOI:** 10.3389/fonc.2025.1689053

**Published:** 2025-10-10

**Authors:** Jing Shang, Yuanyi Xu, Yuejia Tao, Bing Li, Mengqi Li, Jiaxin Guo, Lvjun Yan, Yunning Huang, Qian Ma

**Affiliations:** ^1^ Oncology Hematology Department, University-Town Hospital of Chongqing Medical University, Chongqing, China; ^2^ Third Clinical Medical College, Ningxia Medical University, Yinchuan, China; ^3^ Department of Gastrointestinal Surgery, The Affiliated People’s Hospital of Ningxia Medical University, Yinchuan, China; ^4^ School of Life Sciences, Ningxia University, Yinchuan, China

**Keywords:** dextran sulfate, M2 tumor-associated macrophages, programmed death-ligand 1, proliferation and apoptosis, invasion and migration

## Abstract

**Objective:**

The aim of this study was to investigate the role of dextran sulfate (DS) in M0-to-M2 macrophage polarization and its effect on programmed death-ligand 1 (PD-L1) expression, invasion, migration, proliferation, and apoptosis of human gastric cancer cells (HGCCs) through its action on M2 tumor-associated macrophages (M2-TAMs).

**Methods:**

The effects of DS on M0-to-M2 macrophage polarization and HGCC behavior were examined. CD163 expression was analyzed to determine macrophage polarization, whereas HGCC proliferation, apoptosis, migration, and invasion and PD-L1 expression were quantified. The effect of DS on tumor development was evaluated in an *in vivo* nude mouse model of intraperitoneal implantation by assessing the size and number of implanted nodules. The study also analyzed the association between tumor CD163 and PD-L1 expression.

**Results:**

DS inhibited M0-to-M2 macrophage polarization and HGCC proliferation, invasion, and migration while increasing apoptosis and decreasing PD-L1 expression. DS decreased the number and the size of metastatic tumor nodules in nude mice while decreasing CD163 expression. CD163 expression is positively associated with PD-L1 expression (*p* < 0.01, *R* ^2^ = 0.1613, *N* = 46).

**Conclusion:**

DS inhibits the macrophage transition to the M2 phenotype, leading to a reduced PD-L1 expression and HGCC proliferation, invasion, and migration while increasing cell death.

## Introduction

One of the deadliest tumors worldwide is stomach cancer, often known as gastric cancer (GC) (1), mainly because there is no early diagnosis and no opportunity for surgery (2). More than half of individuals diagnosed with GC ultimately succumb to peritoneal implantation and metastases (3). There is a strong correlation between the tumor microenvironment (TME) and the processes of peritoneal implantation and metastasis in patients with GC. Tumor-associated macrophages (TAMs) are a category of cells that perform essential functions within the surrounding environment of malignant tumors and are intimately connected to the initiation and progression of cancers (4). According to previous reports, the majority of high-density TAMs in tumor tissues are M2 tumor-associated macrophages (M2-TAMs), and a higher proportion of M2-TAMs tends to have a poorer prognosis (5). The surface of cancer cells is a molecular marker for tumors, called programmed death-ligand 1 (PD-L1), which binds to programmed death receptor 1 (PD-1), an immune checkpoint on the surface of cytotoxic T cells that inactivates active T cells that cannot be detected by the immune system and are linked to a poor prognosis (6). The TAM content in tumor tissues is inversely correlated with the PD-L1 expression in cancer cells (7). Therefore, it is vital to determine a solution for the prevention and treatment of GC metastases via intraperitoneal implantation. The identification of novel targets for GC treatment depends strongly on research into how M2-TAMs affect the biologically malignant nature of GC cells.

Dextran sulfate (DS) is widely available in nature, and its biological activity is related to its molecular weight. The project team has been conducting long-term research on the role of the large-molecule DS (molecular weight, 500,000 Da) in intraperitoneal implantation focused on GCs. Previous studies have shown that DS may infiltrate GC cells and exert its effects on the cytoplasm. Moreover, the results of both *in vivo* and *in vitro* experiments have shown that DS can have an impact on GC peritoneal implantation and metastasis through a variety of mechanisms (8, 9). Due to its advantages of slow intraperitoneal absorption, long duration of action, abundant sources, high safety, and low toxicity, macromolecular DS is an improved novel medicine for the treatment of GC implantation and metastasis in the peritoneum; however, its mechanism of action is not well understood and has stymied clinical trials thus far.

In addition to the above-mentioned direct effects, DS may also affect the PD-L1 expression, proliferation, apoptosis, invasion, and migration in human gastric cancer cells (HGCCs) through the indirect effect of the reduction of the number of M2-TAMs via the polarization of M0-TAMs to M2-TAMs, which calls for further research to be conducted. In this work, we investigated the impact of apoptosis and the potential mechanism of DS in HGCCs, specifically how M2 polarization influences the expression of PD-L1 in HGCC, proliferation, invasion, and migration.

## Materials and methods

### Reagents

DS (Sigma, St. Louis, MO, USA) was dissolved in phosphate-buffered saline (PBS) to prepare the stock solution, stored at 4°C, filtered, and sterilized through a 0.22-µm syringe filter. Prior to use, the stock was diluted with culture medium to a final concentration of 0.3%.

### Assessment of the effect of DS on healthy cell viability

The normal human gastric epithelial cell line GES-1 was cultured in RPMI-1640 complete medium until reaching the logarithmic growth phase and then seeded into culture dishes. The control group received no treatment, while the intervention groups were exposed to gradient concentrations of DS (0.1%, 0.2%, and 0.3%) for 24 and 36 h. Cell viability was evaluated using an MTS assay kit. The results indicated that, even at the highest concentration and the longest duration, DS did not cause any significant reduction in cell viability compared with the untreated control group (see **Supplementary Figure S1**).

### Cell lines, nude mice, and human specimens

Zhong Qiaoxiazhou Biotechnology Co., Ltd. provided the human mononuclear cell line THP-1 (ZQ0086), the undifferentiated human GC cell line HGC-27 (ZQ0192), and the well-differentiated human GC cell line AGS (ZQ0240). The short tandem repeat (STR) profiles of the three cell lines are presented in **Supplementary Materials 1**–**3**.

A total of 24 male nude mice (BALb/c, null-colored, 4–6 weeks old, 18–22 g) were used [animal license: SCXX (Ning) 2015-0010]. The mice were housed in a sterile environment with *ad libitum* sterile food/water. The protocol was approved by the Committee for the Ethics of Experiments of Ningxia Medical University (ethics no. IACUC-NYLAC-2019-116).

A total of 46 stomach adenocarcinoma tissue samples (surgically resected and pathologically confirmed) were obtained from Ningxia Hui Autonomous Region People’s Hospital (September 2018–December 2020). None of the patients received preoperative chemo/radiotherapy. All patients provided informed consent. The study was approved by the hospital’s Ethics Committee (2021-NZR010). All procedures involving human samples were conducted in accordance with the ethical standards of the institutional research committee and with the Helsinki Declaration.

### Cell culture

Both AGS and HGC-27 cells were grown in a humidified incubator (37°C, 5% CO_2_) with media (RPMI-1640) supplemented with penicillin–streptomycin (1%) and fetal bovine serum (FBS; 10%). THP-1 cells were cultured in a humidified incubator (37°C, 5% CO_2_) with media (RPMI-1640) supplemented with FBS (10%), penicillin–streptomycin (1%), and β-mercaptoethanol (50 μmol/L).

### Induction and validation of M2 macrophages

At a density of 1 × 10^7^ cells/ml, THP-1 cells were seeded onto 10-mm culture plates during the logarithmic growth phase, and 100 ng/L phorbol 12-myristate 13-acetate (PMA) was added for 48 h. Once that was complete, the control group was given 20 nmol/L of interleukin-4 (IL-4) and IL-13 and the experimental group 20 nmol/L of IL-4, IL-13, and DS for a total of 36 h. The morphological alterations of the THP-1 cells that were stimulated to develop into M2 macrophages were studied under a microscope. The expression of the cell surface marker CD163 antigen was detected using immunofluorescence and Western blotting.

### Quantitative RT-PCR

Using the TRlzol™ Reagent mRNA, total RNA was isolated from each sample. The RNA was then used as a template for cDNA synthesis with a reverse transcription kit, and real-time PCR was carried out with TB Green™ Premix Ex Taq™ II following the guidelines provided by the manufacturer. GAPDH was used in this study as a loading control. The relative mRNA expression was determined using the 2^−ΔΔCT^ approach. All primers used are listed in **Supplementary Table S1**.

### Cell colony formation assay

To assess colony formation, 500 cells were seeded into six-well plates and incubated for 10–13 days, with medium changes taking place every 5 days. Thereafter, the cells were given a thorough washing, fixed with 4% paraformaldehyde, and then stained with 1% crystal violet.

### Nude mouse model of intraperitoneal implantation-induced metastasis

A total of 24 nude mice (BALB/c) were randomized into the control group and the DS group (*n* = 12/group). Each sample received 0.2 ml of HGC-27 cell suspension (5 × 10^7^ cells/ml) via intraperitoneal (i.p.) injection. After 24 h, the DS group received 1 ml of 0.3% DS (i.p.) (10), while the control group received an equal volume of saline. All procedures used strict aseptic techniques. The mice were sacrificed by cervical dislocation on day 14. Metastatic foci on the greater omentum were counted, and the tumor nodule morphology (i.e., size, color, and texture) was recorded. The maximum tumor diameter was ≤15 mm, and the total tumor burden was <5% of the body weight. The omentum with the tumor nodules was processed for further analysis.

### Immunohistochemistry

For mouse tissues, fixed tumor/omentum samples were paraffin-embedded, sectioned (4 μm), and routinely processed (baking, dewaxing, and hydration). For antigen retrieval, sodium citrate buffer was used. The sections were blocked with goat serum to inhibit nonspecific binding and then incubated with primary antibodies (**Supplementary Table S2**) overnight at 4°C. After washing the samples with PBS, secondary antibody incubation (37°C, 30 min) was performed. DAB development, counterstaining, differentiation, dehydration, and clearing were conducted. Brownish-yellow particles indicate PD-L1/CD163 positivity. The slides were mounted with neutral resin. The mean optical density (MOD) was calculated (Image-Pro Plus) from five random fields/section.

For the human GC tissues, dewaxed/hydrated sections were subjected to antigen retrieval (0.2% Triton-100, 15 min), blocked with goat serum (30 min), and incubated with species-matched primary antibodies overnight at 4°C. With appropriate negative controls, fluorescent secondary antibodies were applied and incubated (37°C, 60 min, dark), followed by mounting with DAPI. The localization of PD-L1 (red, cytomembrane) and CD163 (green, cytoplasm) was assessed. The MOD was averaged (Image-Pro Plus) from five random ×400 fields.

### Cellular immunofluorescence

After 15 min of fixation with paraformaldehyde (4%) and 20 min of permeabilization with Triton X-100 (0.2%), the THP-1 cells were cultured on glass coverslips. After blocking the samples for 30 min with 10% goat serum, they were incubated with primary (12 h, 4°C) and secondary (1 h, 37°C) antibodies, after which they were mounted with DAPI. DAPI was used to label the nuclei, while FITC was used to stain the CD163 protein. To calculate the overall image average optical density (MOD), the Image-Pro Plus program was used to select five areas of vision at ×400 magnification at random. In addition, a researcher unaware of the experimental groups quantified the images.

### Western blotting

The cells were lysed, vortexed for 30 s, and incubated on ice (5 min) for five cycles, followed by centrifugation to remove debris. The protein concentration was determined with a BCA assay. The samples were denatured (100°C, 10 min), separated using SDS-PAGE, and then transferred into membranes. The membranes were then blocked, incubated with primary and secondary antibodies (**Supplementary Table S2**), and detected using enhanced chemiluminescence (ECL). The band intensities were quantified (Image-Pro Plus) and normalized to that of β-tubulin.

### Assays for wound healing

The co-culture system was established as follows: During the logarithmic phase of development, the HGC-27 cells were seeded into six-well plates. When they had grown 90%–100% against the plate wall, horizontal lines were drawn between the cells using a 200-μl gun. Thereafter, the cells were treated with PBS three times before being imaged. M2-type macrophages were isolated from cells in the logarithmic growth phase and resuspended in medium to bring their concentration to 5 × 10^5^ cells/ml. The samples were then divided into four different groups: 1) the control group, in which only HGC-27 cells were cultured; 2) the DS group, in which HGC-27 cells were cultured with 0.3% DS; 3) the M2 group, in which Transwells were seeded with M2-type macrophages in the top chamber and HGC-27 cells in the bottom chamber; and 4) the DS+M2 group, in which Transwells were used to cultivate the M2-type macrophages in the upper chamber and the DS-interacting HGC-27 cells in the lower chamber, with the bottom chamber remaining in serum-free media. The process was repeated with another three wells, and then the samples were cultured for 24 and 48 h while pictures were taken and observed under a microscope.

### Assays for Transwell invasion

The matrix gel was diluted and placed in the lower chamber of the top chamber. After the matrix gel had set, the HGC-27 cells were harvested during their logarithmic growth phase and their density set to 5 × 10^4^ cells/ml using FBS-free medium before being injected into the top chamber of the tiny chamber. The lower chambers comprised the following: 1) the control group—complete medium with 10% FBS; 2) the DS group—complete medium with 10% FBS added to 0.3% DS; 3) the M2 group—M2 macrophages grown on full medium containing 10% FBS; and 4) the DS+M2 group—M2-type macrophages cultured with complete medium with 10% FBS added to 0.3% DS. Three wells were used, and the samples were cultured in the incubator for 24 h. The matrix gel and the cells were carefully removed from the top chamber before fixation with paraformaldehyde (4%, 20 min) prior to washing in PBS. For 15 min, they were dyed with 0.1% crystal violet before examination under a microscope. Photographs were taken of five different ×200 areas at random, and the percentage of cells with punctured membranes was determined.

### Statistical analysis

Data are presented as the mean ± SEM, with significance at *p* < 0.05. Two-group comparisons of continuous variables were performed using Student’s *t*-test. For multigroup comparisons, one-way ANOVA with Bonferroni correction was used. Pearson’s correlation was used to assess relationships. Figures were generated using GraphPad Prism 8.0.

## Results

### Induced differentiation and identification of THP-1 cells

**Figure 1:**
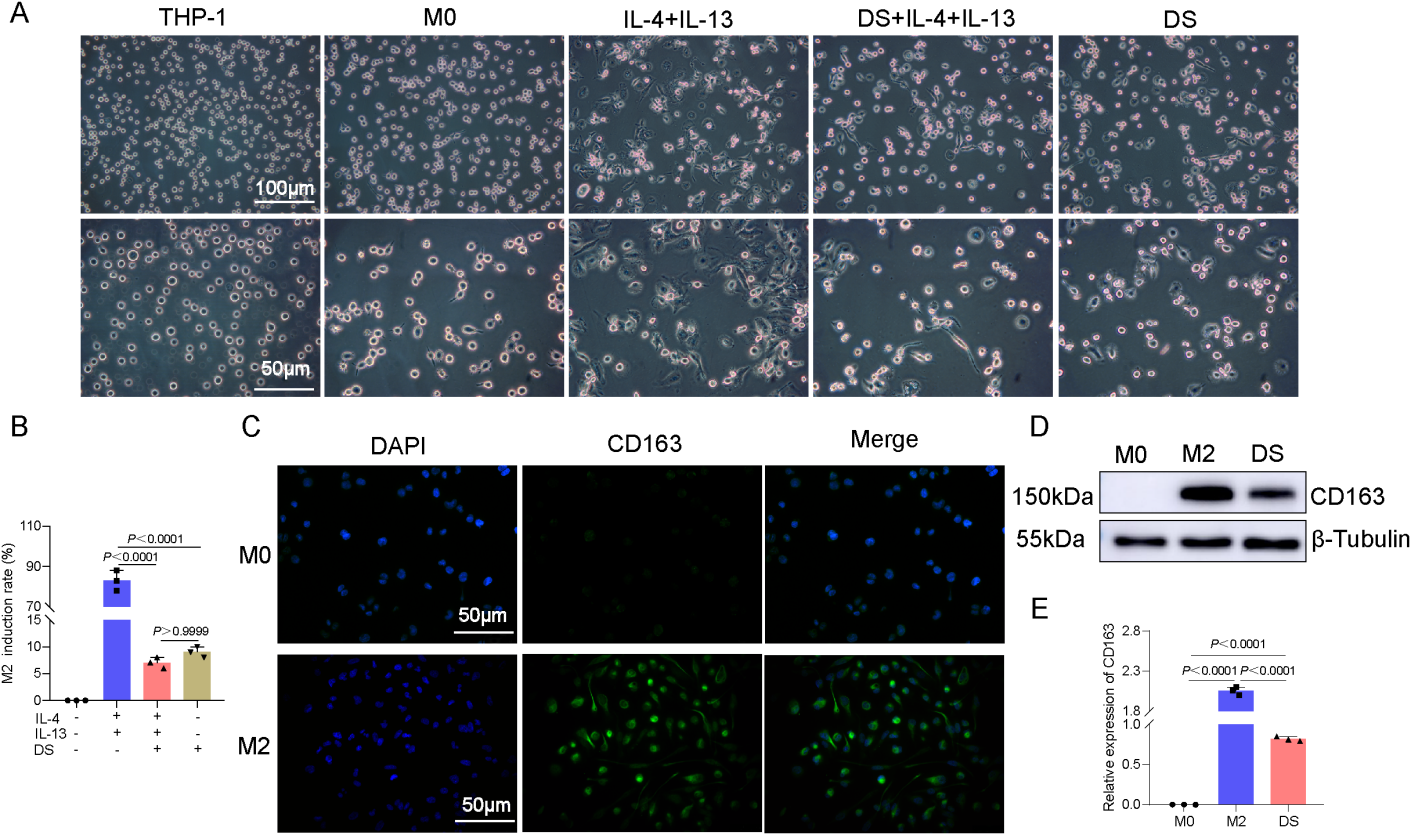
Differentiation of THP-1 monocytes into M0 macrophages induced by phorbol 12-myristate 13-acetate (PMA), identification of macrophages, and effect of dextran sulfate (DS) on the induced differentiation. **(A)** Representative photomicrographs induced under different conditions of THP-1→M0→M2. **(B)** Histogram of the induction rates. **(C)** Cellular immunofluorescence of the protein expression of CD163 molecules in M0 and M2 cells. **(D)** Protein expression bar graphs of CD163 molecules in M0 cells, M2 cells, and DS-intervened (M0→M2) cells. **(E)** Histogram of the Western blot (WB) experiment. Data are presented as the mean ± SEM, and exact *p*-values are shown in the figure panels.

Uninduced THP-1 cells exhibited a spherical morphology. PMA (48 h) induced adherence, cell enlargement, pseudopodia extension, and an M0 macrophage morphology (irregular shape). M0-to-M2 polarization via IL-4/IL-13 (36 h) further increased the cell size, yielding elongated/polygonal cells with extended pseudopodia. Compared with IL-4/IL-13 alone, DS co-treatment (with IL-4/IL-13) or M0-stage DS exposure markedly inhibited the M2 morphology (**Figure 1A**) and reduced its induction rates. Compared with DS+IL-4/IL-13, DS alone did not significantly differ (**Figure 1B**). Cellular immunofluorescence was used for phenotypic identification, and the results revealed that CD163 effectively produced M2-type macrophages that exhibited green fluorescence under a microscope, with the fluorescence being distributed mostly in the cytoplasm and the cytomembrane. The expression of the M2-specific phenotype CD163 was greater than that of the M0 phenotype, suggesting that M2-type macrophages were successfully differentiated by induction (**Figure 1C**). After 36 h of treatment, Western blotting was performed to compare the expression of the M2-specific marker CD163 between two groups: the M2 group (M0 macrophages treated with IL-4 and IL-13) and the DS group (M0 macrophages co-treated with IL-4, IL-13, and DS). Compared with the M0 group, the M2 and DS groups presented significantly higher levels of CD163 protein expression, with the CD163 protein expression being markedly higher in the M2 group than in the DS group (**Figures 1D, E**). The above data indicate that the CD163 expression was elevated in the effectively produced M2-type macrophages and that DS was able to block the polarization of M0 toward M2.

### DS affects the proliferation and apoptotic ability of AGS and HGC-27 cells by affecting the polarization of M2-TAMs

**Figure 2:**
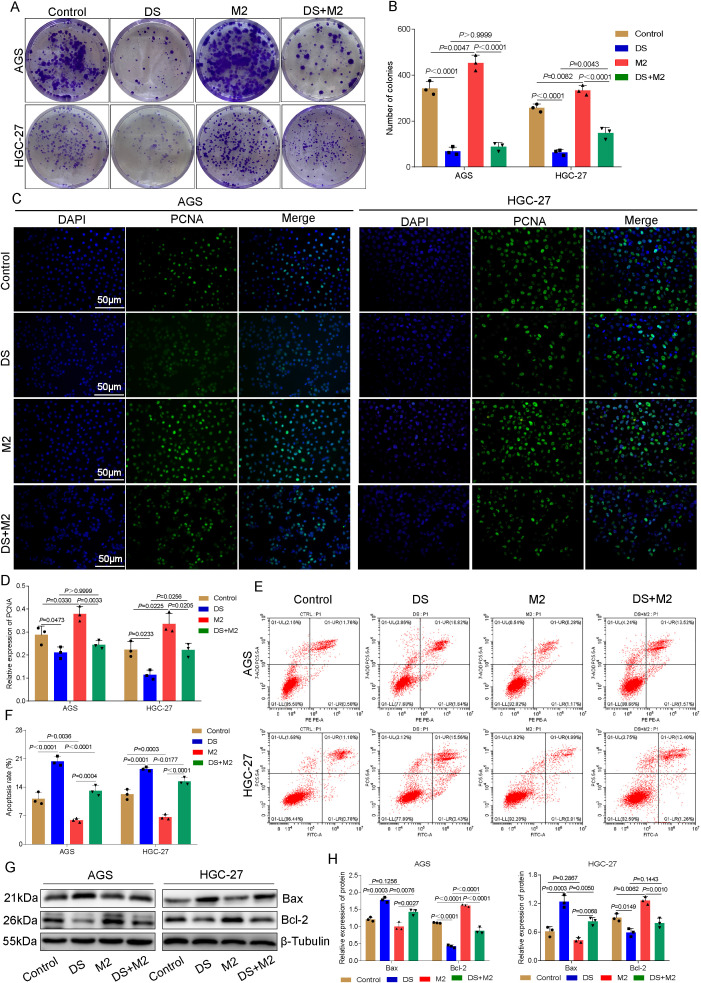
Dextran sulfate (DS) affects the proliferation and apoptotic ability of AGS and HGC-27 cells by acting on the polarization of M2 tumor-associated macrophages (M2-TAMs). **(A)** Plate seeding for the colony formation assay in two cell lines. **(B)** Number of colonies in two cell lines. **(C)** Representative immunofluorescence images of two cell lines after DS treatment. *Scale bar*, 50 μm. **(D)** Relative expression of proliferating cell nuclear antigen (PCNA). **(E)** Flow cytometry analysis of two cell lines. **(F)** Quantitative analysis of the apoptotic rate in two cell lines. **(G)** Western blot bands showing cell proliferation and apoptosis in AGS and HGC-27 cultures treated with DS, M2, and their combination (DS+M2). **(H)** Quantitative analysis of the Western blot results. Data are presented as the mean ± SEM, and exact *p*-values are shown in the figure panels.

Compared with the DS group, a greater proportion of clone spheres formed in the control group, whereas a smaller proportion formed in the DS group compared with the M2 group. On the other hand, there was no statistically significant difference between the DS group and the DS+M2 group, and the number of colonies formed in the DS+M2 group was much smaller than that in the M2 group. In HGC-27 cells, the control group had significantly greater colony formation than the DS group, whereas the M2 group had significantly less colony formation. Clonocyte formation was greater in the DS+M2 group than in the M2 group, whereas it was lower in the DS group (**Figures 2A, B**). The immunofluorescence studies revealed that the green fluorescence of the proliferating cell nuclear antigen (PCNA) protein is indicative of its distribution mostly inside the nucleus. Compared to the DS group, the control group exhibited higher PCNA fluorescence intensity, whereas compared to the M2 group, the control group exhibited lower PCNA fluorescence intensity. There was no statistically significant difference in the PCNA fluorescence intensity between the DS and DS+M2 groups, whereas the intensity in the DS+M2 group was lower than that in the M2 group. For HGC-27 cells, the control group presented greater PCNA fluorescence intensity than the DS group and lower PCNA fluorescence intensity than the M2 group. Compared with that in the M2 group, the PCNA fluorescence intensity in the HGC-27 group was significantly lower; however, the fluorescence intensity in the DS+M2 group was significantly greater than that in the DS group (**Figures 2C, D**). From the flow cytometry measurements, it was revealed that the apoptotic rates for AGS and HGC-27 cells were significantly higher in the DS group compared with the control, but were significantly lower in the M2 group. The results demonstrated that the apoptotic rate was higher in the DS+M2 treatment than in the M2 treatment, whereas it was lower in the DS treatment (**Figures 2E, F**). In AGS cells, Bax had a lower growth in the control group compared with the DS group, while the level in M2 was similar to that in the control group. Compared with the M2 group, the DS+M2 group presented a higher Bax, but a lower Bax than the DS group. Bcl-2 was higher in the control group than in the DS group and was significantly greater in the M2 group than in the control group. The DS+M2 group presented a lower Bcl-2 compared with the M2 group, but a higher Bcl-2 compared with the DS group. In HGC-27 cells, Bax was significantly lower in the control group than in the DS group and was further reduced in the M2 group compared with the control group. The DS+M2 macrophages presented higher Bax levels compared with the M2 macrophages, but had lower Bax levels than the DS-treated macrophages. Bcl-2 was significantly elevated in the control group compared with the DS group and increased in the M2 group compared with the control group. Compared with M2, DS+M2 resulted in reduced Bcl-2 (**Figures 2G, H**). DS suppresses HGCC proliferation and weakens the capacity of M2 macrophages to prevent HGCC apoptosis, as shown by the data above. DS promotes apoptosis, but suppresses HGCC growth, while M2 promotes HGCC proliferation, but blocks HGCC apoptosis.

### DS reduces the capacity of HGC-27 cells to invade and migrate by altering the polarization of M2-TAMs

**Figure 3:**
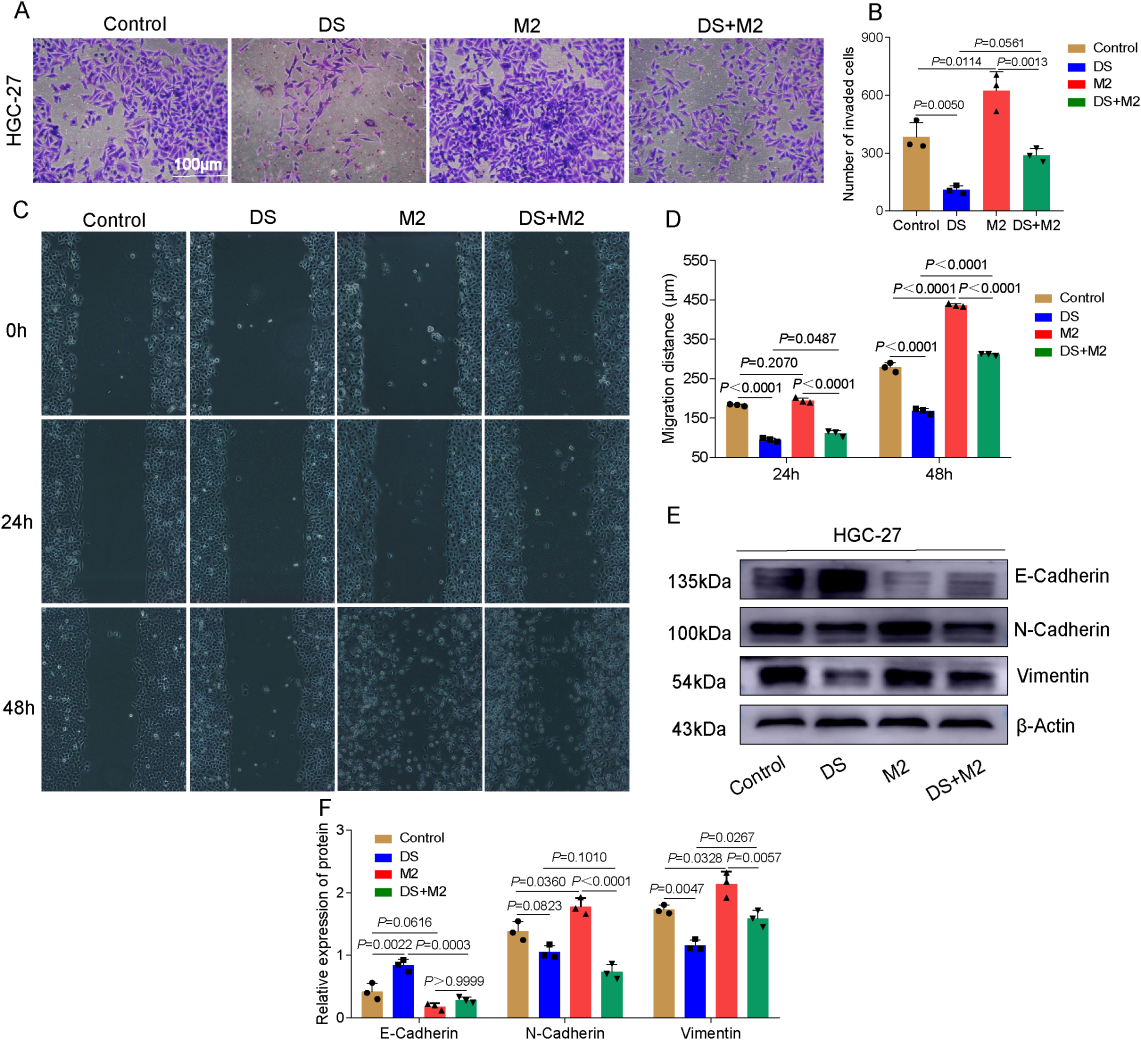
Dextran sulfate (DS) reduces the capacity of HGC-27 to invade and migrate via altering the polarization of M2 tumor-associated macrophages (M2-TAMs). **(A)** Representative images of the Transwell invasion assay in HGC-27 cells. *Scale bar*, 100 μm. **(B)** Quantitative analysis of the number of invaded cells among different groups. **(C)** Representative images of the wound healing assay. **(D)** Quantitative analysis of the migration distance among different groups. **(E)** Western blot bands showing the invasiveness of HGC-27 cells as a function of exposure to DS, M2, and their combination (DS+M2). **(F)** Quantitative analysis of the Western blot. Data are presented as the mean ± SEM, and exact *p*-values are shown in the figure panels.

The results of the Transwell invasion experiment revealed that the number of cells able to penetrate the membrane in the DS group was significantly lower than that in the control group, whereas the number of cells able to penetrate the membrane in the M2 group was significantly greater. The number of cells able to traverse the membrane in the M2 group was significantly greater than that in the DS+M2 group, while the number of cells able to traverse the membrane in the DS group was significantly lower than that in the DS+M2 group (**Figures 3A, B**). The results revealed statistically significant differences among multiple groups, indicating differences in the migration distance between groups. According to the findings of the additional pairwise comparison, throughout the 24-h duration, the migration distance of the control was greater than that of the DS; however, the migration distance of M2 was shorter. Although the migration distance of the DS+M2 group was less than that of the M2 group, the migration distance of this group was greater than that of the DS group. Those in the control treatment migrated farther after 48 h compared with those in the DS treatment, whereas those in the M2 treatment migrated farther than those in the control. However, when the DS group was compared with the control and M2 groups in terms of the average distance migrated, a statistically significant difference was detected. The migration distance was reduced in the DS+M2 treatment compared with the M2 treatment, but it was greater for the DS+M2 treatment than for the DS treatment (**Figures 3C, D**). Western blotting experiments revealed that the protein expression of E-cadherin was considerably lower in the control group than in the DS group and was significantly lower in the M2 group than in the control group. Nonetheless, it was expressed at a much higher level in the DS group. The protein expression of N-cadherin was significantly greater in the control group than in the DS group; however, its expression was markedly lower than that in the M2 group. The N-cadherin protein expression was considerably lower in the DS+M2 group than in both the M2 and DS groups. The vimentin protein expression was markedly increased in the control group compared with the DS group, whereas it was markedly lower in the control group than in the M2 group. The vimentin protein expression in DS+M2 treatment was significantly lower than that in the M2 treatment, although it was much higher than that in the DS treatment (**Figures 3E, F**). Based on the findings presented above, the potential of HGCCs to invade new territories and migrate may be hindered by DS, whereas these can be stimulated by M2. The capability of M2 to invade and migrate to promote HGCCs may be blocked by DS.

### DS inhibits the expression of PD-L1 in AGS and HGC-27 cells through modulating the polarization of M2-TAMs

**Figure 4:**
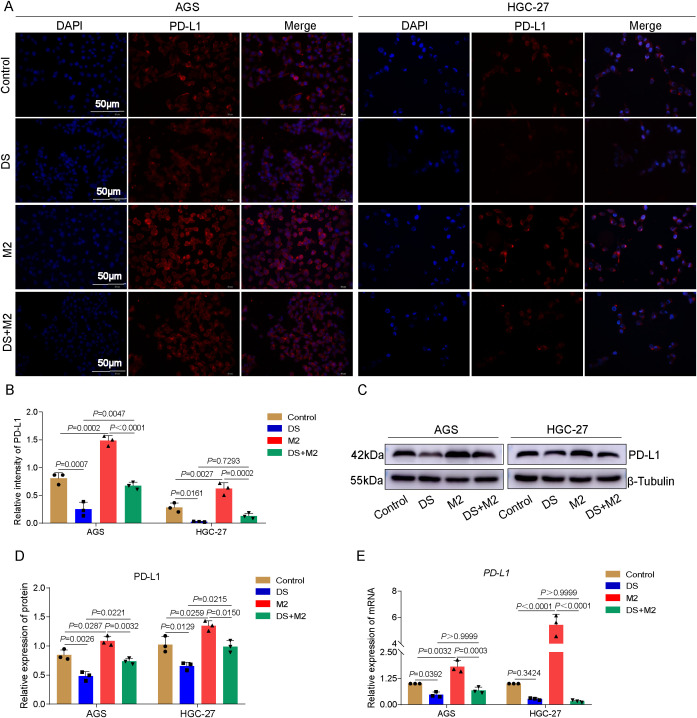
Dextran sulfate (DS) inhibits the expression of programmed death-ligand 1 (PD-L1) in AGS and HGC-27 cells through modulating the polarization of M2 tumor-associated macrophages (M2-TAMs). **(A)** Representative immunofluorescence images of two cell lines. *Scale bar*, 50 μm. **(B)** Quantitative analysis of the relative PD-L1 intensity in two cell lines. **(C)** Representative Western blot of PD-L1 protein expression induced by DS, M2, and their combination (DS+M2) in AGS and HGC-27 cells. **(D)** Quantitative analysis of the Western blot. **(E)** qRT-PCR analysis of PD-L1 mRNA expression induced by the same treatments in AGS and HGC-27 cells. Data are presented as the mean ± SEM, and exact *p*-values are shown in the figure panels.

The results presented in this study demonstrated that red fluorescence was caused by PD-L1 protein expression, which was mostly localized in the cytoplasm and the cytomembrane. It was found that the PD-L1 fluorescence intensity of the control group was much higher than that of the DS group, but was markedly lower than that of the M2 group. These findings pertain to AGS cells. In the group that was given both DS and M2, the fluorescence intensity of PD-L1 markedly decreased, but the fluorescence intensity in this group was much greater than that of the DS group. The control group had greater PD-L1 fluorescence intensity than the DS group, but a lower PD-L1 fluorescence intensity than the M2 group. These results were obtained from the HGC-27 experiment. There was no statistically significant difference in the PD-L1 fluorescence intensity between the DS and DS+M2 groups. Compared with the DS+M2 group, the M2 group presented significantly greater PD-L1 fluorescence intensity (**Figures 4A, B**). The results also revealed that the PD-L1 protein expression level in the control AGS and HGC-27 cells was higher than that in DS cells, although it was weaker than that in M2 cells in terms of intensity. As shown in **Figures 4C, D**, the PD-L1 protein was expressed at a lower level in the DS+M2 group than in the M2 group and was also expressed at a lower level in the DS group than in the DS+M2 group. The results of the qRT-PCR experiments revealed that the expression of the PD-L1 mRNA in the AGS and HGC-27 cells in the control group was greater than that in the DS group, but was lower than that in the M2 group. **Figure 4E** shows statistically significant differences in the PD-L1 mRNA expression between the DS+M2 and M2 macrophages. These findings suggest that DS can inhibit the expression of PD-L1 in HGCCs, whereas M2 can do the opposite. DS can inhibit the expression of PD-L1 in HGCCs promoted by M2.

### Effect of DS on HGCCs with intraperitoneal implantation metastasis

**Figure 5:**
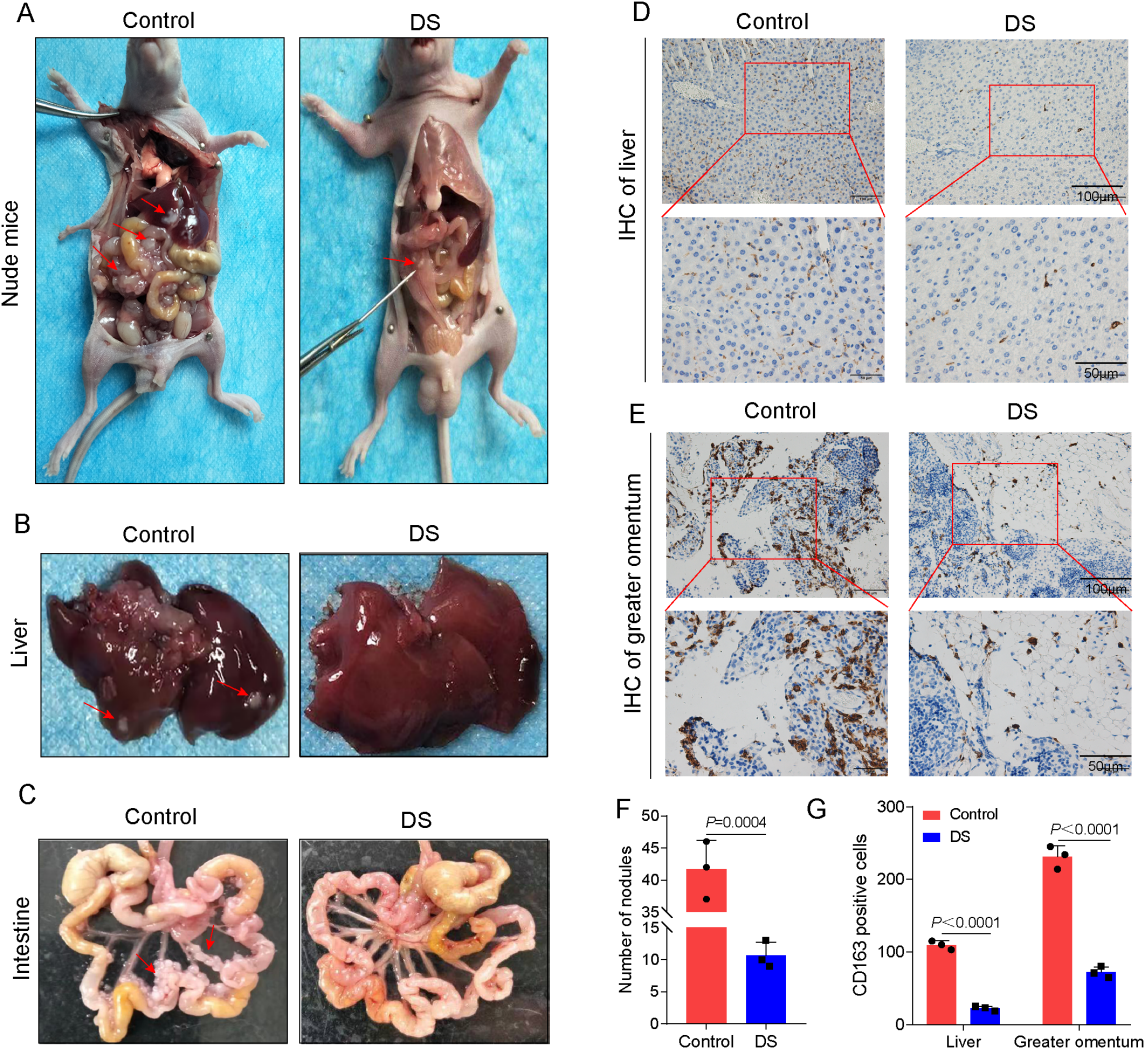
Effect of dextran sulfate (DS) on human gastric cancer cells (HGCCs) with intraperitoneal implantation metastasis. **(A)** Gross appearance of the peritoneal tumor nodules in nude mice. **(B)** Gross histopathological changes in the liver. **(C)** Gross morphological changes in the intestine. **(D)** Representative immunohistochemical images of the liver tissue. **(E)** Representative immunohistochemical images of the omental tissue. *Scale bars*, 50 and 100 μm. **(F)** Quantitative analysis of the number of nodules. **(G)** Quantitative analysis of the CD163-positive cell expression in tumor tissues of nude mice and on the intraperitoneal implantation of gastric cancer (GC) metastatic tumors. Data are presented as the mean ± SEM, and exact *p*-values are shown in the figure panels.

Metastases from the intraperitoneal implantation of HGC-27 cells showed a firm whitish gray hue. Both the size and the number of nodules formed by the intraperitoneal implantation of GC cells were considerably lower in the DS group compared with the control group (**Figures 5A–C, F**). Immunohistochemistry studies were carried out to determine the impact of DS on the expression of CD163 in the liver and larger omentum tissues, which are representative of peritoneal implantation metastases in nude mice. A positive CD163 expression was observed to be localized in the cytoplasm and membrane and to have a brownish color in the DS group, and the DS group had a much lower number of CD163-positive cells in the liver and larger omentum tissues compared with the control (**Figures 5D, E, G**). The above results suggest that DS significantly inhibited the intraperitoneal implantation and metastasis of HGC-27 cells. DS can dramatically downregulate the expression level of CD163 in more omentum and liver metastases. Therefore, DS may inhibit the proliferation and intraperitoneal implantation of HGC-27 cells by affecting the polarization of M0 macrophages toward M2-TAMs in nude mice, reducing the number of M2-TAMs.

### Differentiated GCs express PD-L1 and CD163 at varying levels

**Figure 6:**
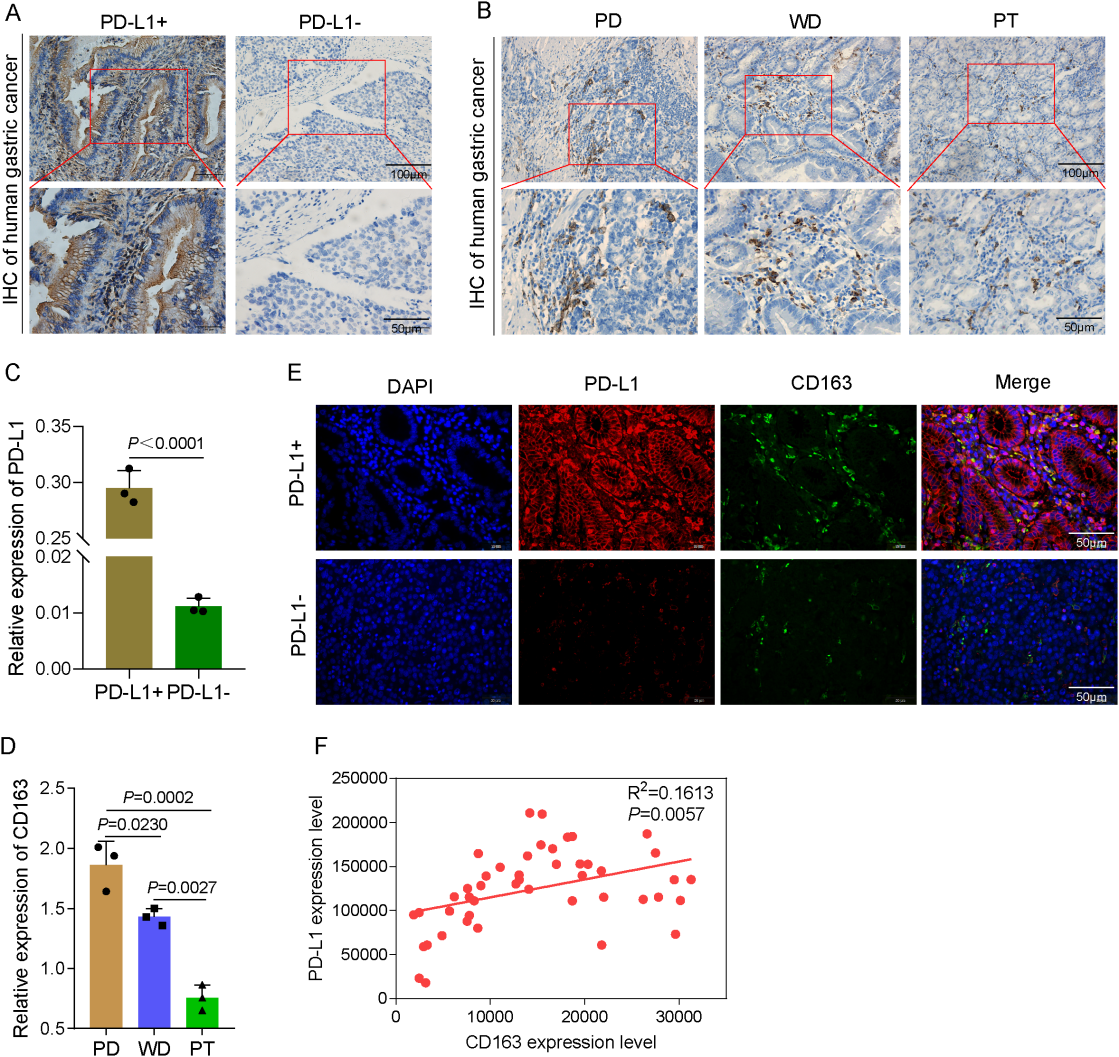
Differentiated gastric cancer (GC) express programmed death-ligand 1 (PD-L1) and CD163 at varying levels. **(A)** Representative immunohistochemical images of human GC tissues. *Scale bars*, 50 and 100 μm. **(B)** Representative immunohistochemical images of poorly differentiated (PD) GC, well-differentiated (WD) GC, and paracancerous tissues (PT). **(C)** Quantitative analysis of the relative PD-L1 expression. **(D)** Quantitative analysis of the relative CD163 expression. **(E)** Immunofluorescence to detect PD-L1 and CD163 expression in several types of differentiated GC. **(F)** Analysis of the correlation between PD-L1 and CD163 expression levels. Data are presented as the mean ± SEM, and exact *p*-values are shown in the figure panels.

The expression of PD-L1- and M2-TAM-specific phenotypic CD163 proteins in GC tissues and paraneoplastic tissues was examined using immunohistochemical labeling. The results of the present study demonstrated that the PD-L1-positive expression was restricted to the cytoplasm and the cell membrane, with the latter showing a mostly brownish yellow hue. The protein expression level of PD-L1 in the PD-L1-positive (PD-L1+) group was considerably greater compared with that in the PD-L1-negative (PD-L1−) group (**Figures 6A, C**). When the CD163 protein was expressed in GC tissues with varying degrees of differentiation, the positive CD163 expression was restricted to the cytoplasm and the cell membrane, and the expression was more evenly distributed in the tumor mesenchyme (**Figure 6B**). As in both well-differentiated (WD) and poorly differentiated (PD) adenocarcinomas, the protein expression of CD163 was considerably greater than that in paraneoplastic tissues (PT). The CD163 protein expression level in the PD group was significantly greater compared with that in the WD group (**Figure 6D**). These data imply that M2-TAM infiltration is positively related to the degree of malignancy of GC clinicopathological differentiation, with greater levels of malignancy associated with more M2-TAM infiltration in HGC tissues. Double-labeling immunofluorescence revealed the PD-L1 and CD163 expression. Merged imaging revealed green fluorescence localized to the tumor mesenchyme and red fluorescence to the tumor parenchyma. The degree of positive expression of PD-L1 was significantly associated with the degree of positive expression of CD163 (**Figures 6E, F**). Based on the aforementioned findings, the rate of M2-TAM infiltration in the mesenchyme of HGC tumor cells was closely related to the expression of PD-L1, suggesting that M2-TAMs may upregulate the PD-L1 expression in HGCCs. These results bridge our mechanistic data with clinical relevance, suggesting that the axis targeted by DS has clinical significance.

## Discussion

The two primary causes of mortality for people with stomach cancer are recurrence and metastasis, and tumor molecular markers are significant in determining the prognosis and predicting tumor recurrence (11). Removing micrometastases and lingering tumors after surgical metastases in the case of GC is an efficient therapeutic method (12). The majority of intravenous chemotherapeutic drugs cannot reach the peritoneal cavity, resulting in a reduced efficacy; therefore, drugs injected directly into the peritoneal cavity are more likely to reach effective concentrations. DS is a potential drug with slow absorption in the peritoneal cavity, a long duration of action, high safety, and low toxicity.

Local hypoxia, inflammation, and elevated lactate levels are some of the microenvironmental alterations that occur alongside tumor growth. These changes are caused by the secretion of chemokines, cytokines, and other substances by tumor cells, mesenchymal cells, and immune cells (13, 14). Furthermore, a number of blood monocytes are attracted into the TME as TAMs (15), where they broadly differentiate into two classes with distinct immunological defense and immune surveillance roles. Specifically, one type comprises the classically activated macrophages (M1), which are pro-inflammatory and antitumor factors that prevent the immune escape of tumors. The other type comprises the alternatively activated macrophages (M2), which have pro-tumor and anti-inflammatory effects associated with the tumor immune escape capacity (16, 17). Therefore, M2-TAMs play a role in encouraging invasive metastasis and dissemination in the TME of advanced GC, which is a significant factor contributing to its poor prognosis (18). This is one of the reasons for the poor prognosis of this type of cancer. It was hypothesized, on the basis of previous research, that DS, in addition to its direct effect on HGCCs, also inhibits the peritoneal implantation and metastasis of GC. This would be accomplished by reducing the indirect effect of M2-TAM production in the intratumor microenvironment, which would in turn reduce the biological activity of tumor cells. However, the precise molecular process is not entirely clear.

Western blotting was used in this investigation to determine the impact of DS on M0-induced differentiation. The result is consistent with the morphological finding that DS can inhibit the polarization of M0 toward M2, and the mechanism may involve DS acting on the cytokines IL-6, IL-10, and TGF-β to activate M2 polarization and the monocyte colony-stimulating factor or other signaling pathways that affect the polarization of M2-type macrophages (19, 20). Although the TAMs in the TME are dominated by M2 macrophages (21), an *in vitro* model utilizing PMA to differentiate human THP-1 monocytes into M0 macrophages and subsequently polarize them to an M2 phenotype can recapitulate a similar cellular phenotype and, to a certain extent, mimic the pro-tumorigenic function of the M2-TAMs observed *in vivo*.

In this work, colony formation and cellular immunofluorescence were utilized to measure the proliferation capacity of cells in order to further study the effects of DS on the ability of HGCCs to proliferate via M2 polarization. The results revealed that the application of DS considerably reduced the number of colonies formed and the intensity of the PCNA fluorescence, suggesting that DS may effectively suppress the proliferation of HGCCs. The number of clones formed and the fluorescence intensity of PCNA increased significantly after co-culture with M2, indicating that M2 can significantly promote the proliferation ability of HGCCs. Following co-culture with M2 and DS, the number of clones formed and the fluorescence intensity of PCNA dramatically decreased, indicating that DS may considerably restrict the capacity of M2 to proliferate and promote HGCCs. The apoptotic capacity of each group was determined using flow cytometry and Western blot in order to investigate the impact of DS on the ability of HGCCs to promote apoptosis by affecting the polarization of M2. After DS intervention, the results revealed an increase in the apoptotic rate, a large increase in the expression of the Bax protein, and a considerable decrease in the expression of the Bcl-2 protein, demonstrating that DS may considerably boost the potential of HGCCs to undergo apoptosis. After co-culturing with M2, the rate of apoptosis decreased, the protein expression of Bcl-2 considerably increased, and the protein expression of Bax greatly decreased, indicating that M2 may strongly suppress the capacity of HGCCs to undergo apoptosis. After co-culturing with DS-treated M2 macrophages, the rate of apoptosis greatly increased, the protein expression of Bcl-2 significantly decreased, and the protein expression of Bax significantly increased, indicating that DS significantly attenuated the ability of M2 macrophages to inhibit the apoptosis of HGCCs. Transwell, scratch, and Western blot assays were then utilized to determine the ability of the cells to invade and migrate and how DS affects M2 polarization and the migration and invasion ability of HGC-27 cells. The results of the invasion and migration experiments were consistent. After DS intervention, there was a significant reduction in the number of cells that penetrated the membrane, a significant decrease in the migration distance, a significant increase in the protein expression of E-cadherin, and a significant decrease in the protein expression of N-cadherin and vimentin. These results suggest that DS may suppress the ability of HGC-27 cells to invade and migrate. In addition to increasing the number of cells that were able to penetrate the membrane after co-culturing with M2, it also increased the migration distance, decreased the protein expression of E-cadherin, and increased the protein expression of N-cadherin and vimentin. These results suggest that M2 may enhance the invasion and migration ability of HGC-27 cells. Following co-culture with DS-intervened M2, the proportion of membrane-penetrating cells was dramatically reduced, the migratory distance was shortened, and E-cadherin was shown to have considerably higher expression levels. Indicating that DS may be able to block the capacity of M2 macrophages to invade and migrate to promote HGC-27 cells, the C-cadherin expression was dramatically increased, whereas the N-cadherin and vimentin protein expression was significantly reduced. Based on the aforementioned findings, DS may effectively stop HGCCs from proliferating, invading, and migrating, promoting cell death. M2 macrophages may encourage HGCCs to proliferate, invade, and migrate while simultaneously reducing their capacity to undergo apoptosis. Conversely, DS might counteract the ability of M2 to encourage HGCCs to proliferate, invade, and migrate while attenuating its ability to prevent apoptosis. We may thus use the investigation on the mechanism related to DS intervention in which M0 macrophages block their polarization to the M2 phenotype as the primary research topic for our subsequent experiments.

Nude mice with tumor nodules implanted in their peritoneal cavity showed a significant increase in volume, which increased significantly as the 36-hour duration in HGC-27 cells was extended, according to *in vivo* animal research. Compared with the control group, the DS group was able to stop HGCCs from proliferating, from being implanted intraperitoneally, and from metastasizing. Previous studies reported that human GC is prone to liver metastasis and peritoneal implantation metastasis (22). Therefore, the liver and greater omentum were chosen as the main sites of observation. According to the immunohistochemical findings, DS may be able to lower the number of M2-TAMs by inhibiting the expression of CD163 in hepatic and omental metastases. Tumor exosomes are involved in the omental metastatic colonization of GC by inducing adaptive responses in the TME (23), and they may be the main cause of the elevated expression levels of CD163. It was inferred that DS might inhibit the proliferation, abdominal implantation, and metastasis of HGC-27 human GC cells by affecting the polarization of M0 macrophages toward M2-TAMs and by reducing the number of M2-TAMs in nude mice *in vivo*. DS is widely used in murine models of inflammatory bowel disease (IBD). We ensured careful control over the injection method to minimize any nonspecific side effects that may occur. In addition, we closely monitored the animals for signs of peritonitis following intraperitoneal injection. By using appropriate intraperitoneal dosages, we implemented risk mitigation strategies to ensure the safety and wellbeing of the animals in our study.

Numerous cancer types are now being treated with increasing frequency via vaccination. Immune checkpoint proteins such as PD-L1 may be upregulated by cancer cells to help avoid immune monitoring (24). Immune checkpoint blockade (ICB), such as pembrolizumab and nivolumab, which are capable of inhibiting PD-1 and PD-L1 from interacting with one another, may be used to prevent this escape (25). Within the context of this experiment, the M2 macrophages were co-cultured with AGS and HGC-27 in order to evaluate their influence on the expression of PD-L1 in HGCCs. M2 macrophages were induced by THP-1 cells. The results revealed that the protein and mRNA expression levels of PD-L1 in HGCCs decreased following DS intervention, indicating that DS might block PD-L1 expression in HGCCs. Cellular immunofluorescence and qRT-PCR were employed to determine the PD-L1 expression level in each set of cells. The expression of PD-L1 was upregulated at the protein and mRNA levels in cells co-cultured with M2 macrophages, indicating that M2 macrophages may increase PD-L1 expression in HGCCs. The expression of PD-L1 was downregulated in cells co-cultured with M2 that received intervention with DS, suggesting that DS could inhibit the expression of PD-L1 in M2-promoted HGCCs. Using immunohistochemistry and immunofluorescence, we analyzed the expression of PD-L1 and CD163 in clinical GC samples and found that the degree of infiltration of M2-TAMs may be related to clinicopathological features: the greater the degree of pathological differentiation and malignancy, the greater the number of M2-TAMs in the GC microenvironment. PD-L1 expression correlated with CD163-positive macrophage infiltration, suggesting that M2-TAMs may promote PD-L1 expression in human GC tumor cells. It has been reported in the literature that the infiltration of M2-TAMs is significantly associated with poor prognosis, progression, and other adverse clinical outcomes in a wide range of cancers (26, 27); however, M2-TAMs can also inhibit the immunotherapeutic effect by suppressing T-cell activity and enhancing the PD-L1 expression in the TME during anti-PD-1/PD-L1 immunotherapy (28–30). This clinical correlation strengthens the biological significance of our findings and supports the potential therapeutic relevance of targeting the macrophage–tumor cell interaction with DS.

Although the current study provides a solid mechanistic foundation for the antitumor effects of DS, several limitations should be acknowledged. Firstly, the experimental models relied on established cell lines and THP-1-induced macrophages. Validation using patient-derived organoids and primary human macrophages—particularly those derived from the peritoneal cavity—would be valuable to further confirm the translational potential of DS. Secondly, this study primarily focused on elucidating the biological effect of DS on peritoneal metastasis rather than conducting a full therapeutic efficacy trial. Future studies incorporating detailed survival analysis and comprehensive toxicity evaluation will be essential next steps in the translational pathway of DS. In addition, the use of orthotopic or systemic metastasis models could help further assess the impact of DS on organotropic metastasis to sites such as the liver and the spleen. These aspects will be emphasized in our subsequent investigations to promote the clinical application of DS.

The TME guides macrophage polarization through metabolic signaling, in which DS has emerged as a potent inhibitor of the M2 phenotype. Our results showed that DS treatment dampened PD-L1 expression; suppressed HGCC proliferation, invasion, and migration; and promoted cell death, consistent with a broad antitumor response. Unlike pro-M2 metabolites such as lactate, which fuels the tricarboxylic acid (TCA) cycle to promote adenosine triphosphate–citrate lyase (ACLY)-dependent histone acetylation and M2 gene expression (31), or sarcosine, which enhances the anti-inflammatory macrophage polarization via the general control nonderepressible 2 (GCN2) signaling pathway (32), DS acts as a disruptor of M2 polarization. Similarly, while itaconate suppresses M2 polarization via direct covalent inhibition of Janus kinase 1 (JAK1) (33), we propose that DS operates through a distinct upstream mechanism, potentially interfering with critical receptor–ligand interactions or endocytic processes required for M2 signal transduction. This prevents the metabolic reprogramming and epigenetic changes necessary for PD-L1 upregulation and functional M2 polarization. Thus, DS represents a unique macromolecular strategy that uncouples the TME from immunosuppressive macrophage activation, acting independently of metabolic substrate utilization or kinase inhibition.

In conclusion, M2 macrophages may increase the expression of PD-L1 in HGCCs, whereas DS macrophages can decrease the expression of PD-L1 in M2 macrophages while increasing HGCCs, hence decreasing cancer’s ability to evade the immune system. M2 promotes the proliferation, invasion, and migration in HGCCs, thereby weakening its own pro-apoptotic effect and inhibiting HGCC cell death. DS can inhibit this promoting effect of M2. The suppression of the polarization process from M0 to M2 decreases the number of M2 macrophages and thus impacts tumor growth, apoptosis, invasion, and migration, which may be the mechanism involved. This research provides a fresh foundation for the development of potent medications for the prevention and management of GC abdominal metastasis.

## Data Availability

The datasets presented in this study can be found in online repositories. The names of the repository/repositories and accession number(s) can be found in the article/**Supplementary Material**.

## Supplementary material

Supplementary Figure 1
**|** Effect of various concentrations of DS on the viability of GES-1 cells. Data were presented as mean ± S.E.M. and exact *P* value were shown in figure panels.
