# Supplementary material for: Dextran sulfate inhibits the invasion, migration, and programmed death-ligand 1 expression in human gastric cancer cells by affecting the M2 tumor-associated macrophage polarization
Source: Front Oncol. 2025 Oct 10;15:1689053. doi: 10.3389/fonc.2025.1689053 (PMC12549276; doi:10.3389/fonc.2025.1689053)
Supplement: Supplementary file 1 [file Table1.docx]

**Supplementary table1 Gene-specific sequences**

| Gene | Species | Forward primer sequence(5'-3') | Reverse primer sequence(5'-3') |
| --- | --- | --- | --- |
| *PD-L1* | Mus musculus | TGTTGAAGGACCAGCTCTCCC | ATCATGCAGCGGTACACCCC |
| *GAPDH* | Mus musculus | CAGGAGGCATTGCTGATGAT | GAAGGCTGGGGCTCATTT |

**Supplementary table 2 Antibodies and dilution ratios**

| Antibodies | Source | Mol.Wt | Dilution ratio | Identifier |
| --- | --- | --- | --- | --- |
| CD163 monoclonal antibody | Rabbit | 150 | 1:1000 | ab182422 |
| PD-L1 monoclonal antibody | Mouse | 42 | 1:1000 | ab238697 |
| Bax polyclonal antibody | Rabbit | 21 | 1:500 | ab53154 |
| Bcl-2 polyclonal antibody | Rabbit | 26 | 1:1000 | ab196495 |
| Vimentin monoclonal antibody | Rabbit | 54 | 1:1000 | ab92547 |
| N Cadherin polyclonal antibody | Rabbit | 100 | 1:2000 | ab18203 |
| E Cadherin monoclonal antibody | Rabbit | 135 | 1:5000 | ab76319 |
| Anti β-Actin monoclonal Antibody | Mouse | 43 | 1:5000 | CW0098M |
| [Anti β-Tubulin monoclonal Antibody](https://www.cwbio.com/product/detail/id/10115) | Mouse | 55 | 1:5000 | CW0096M |
| HRP-conjugated Affinipure Goat Anti-Mouse IgG(H+L) | Mouse | - | 1:10000 | SA00001-1 |
| Goat anti-Rabbit IgG (H+L) Antibody, HRP | Rabbit | - | 1:5000 | 31460 |
