# Supplementary material for: Dextran sulfate inhibits the invasion, migration, and programmed death-ligand 1 expression in human gastric cancer cells by affecting the M2 tumor-associated macrophage polarization
Source: Front Oncol. 2025 Oct 10;15:1689053. doi: 10.3389/fonc.2025.1689053 (PMC12549276; doi:10.3389/fonc.2025.1689053)

**Supplementary Materials 1 STR Profiling of the Undifferentiated Human Gastric Cancer (GC) Cell Line HGC-27**


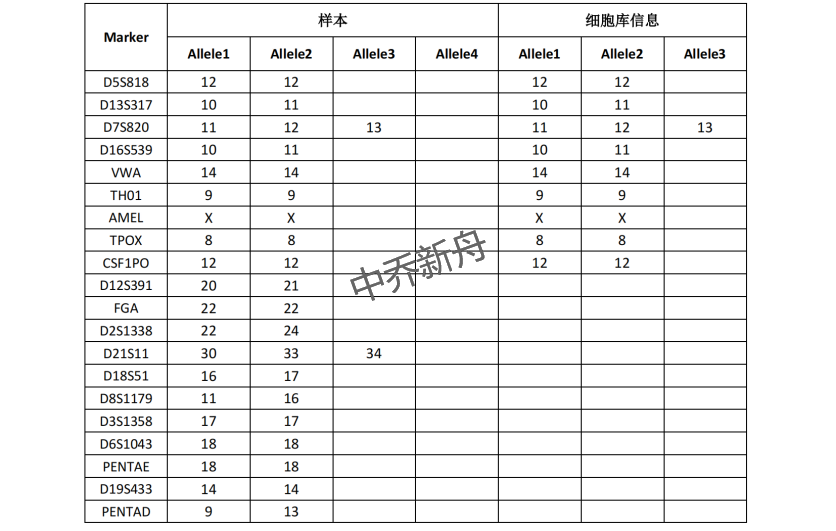


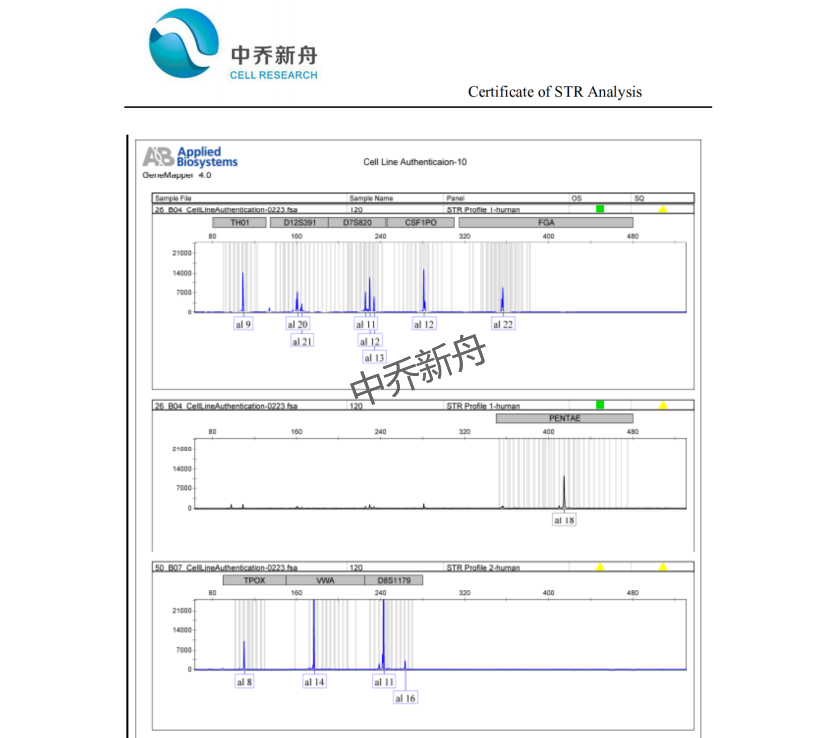


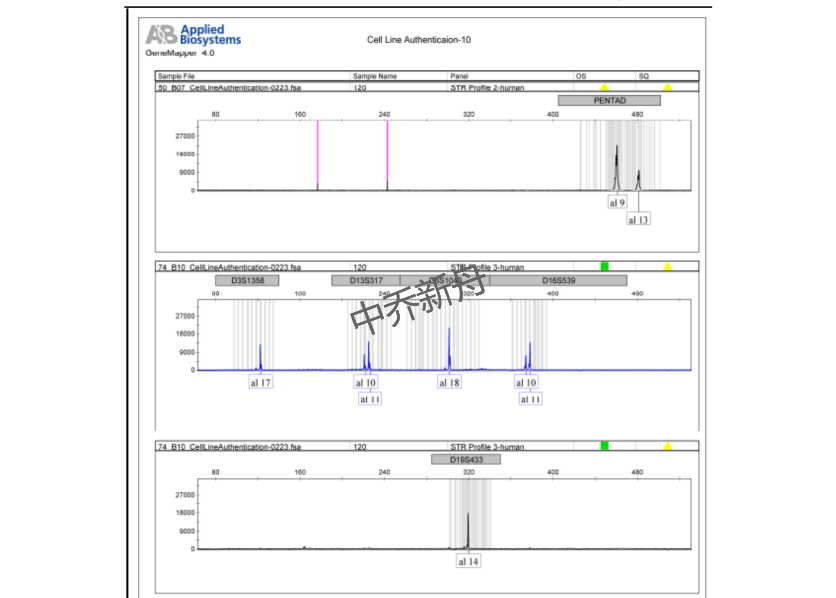


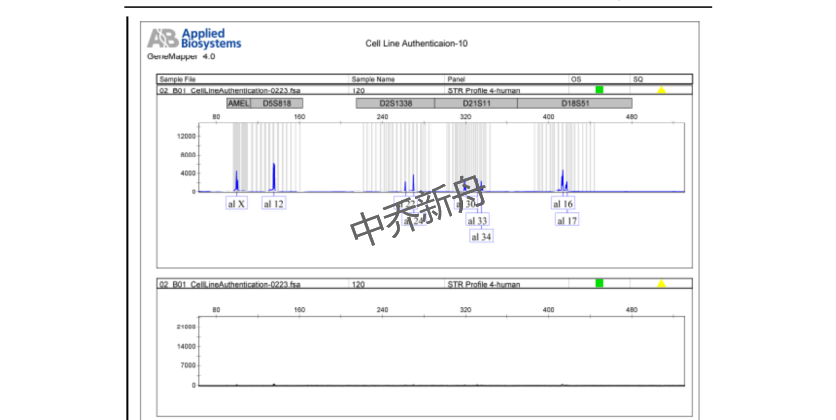


**Supplementary Materials 2 STR Profiling of the well-differentiated human GC cell line AGS**


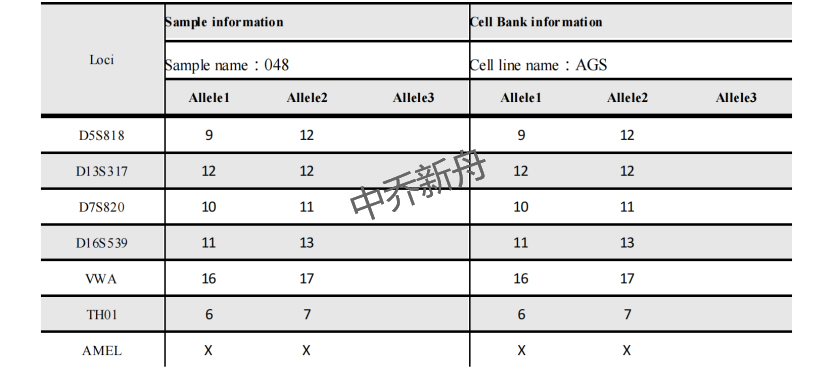


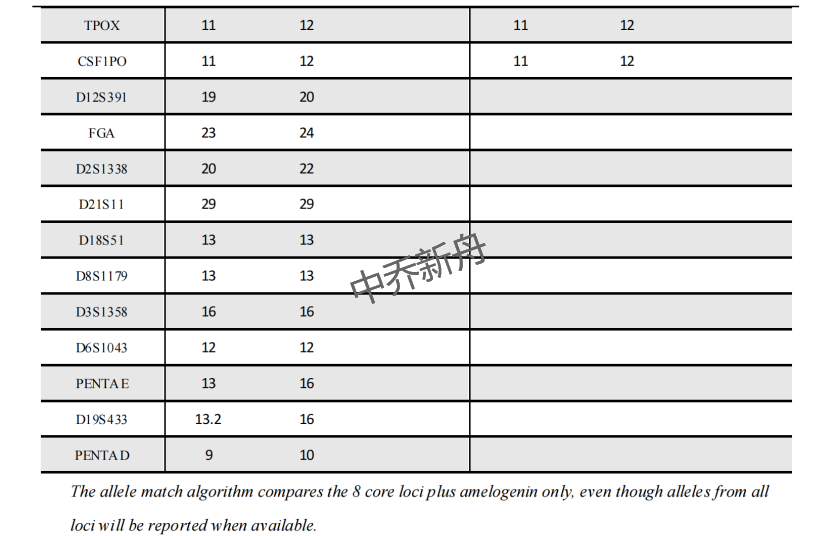


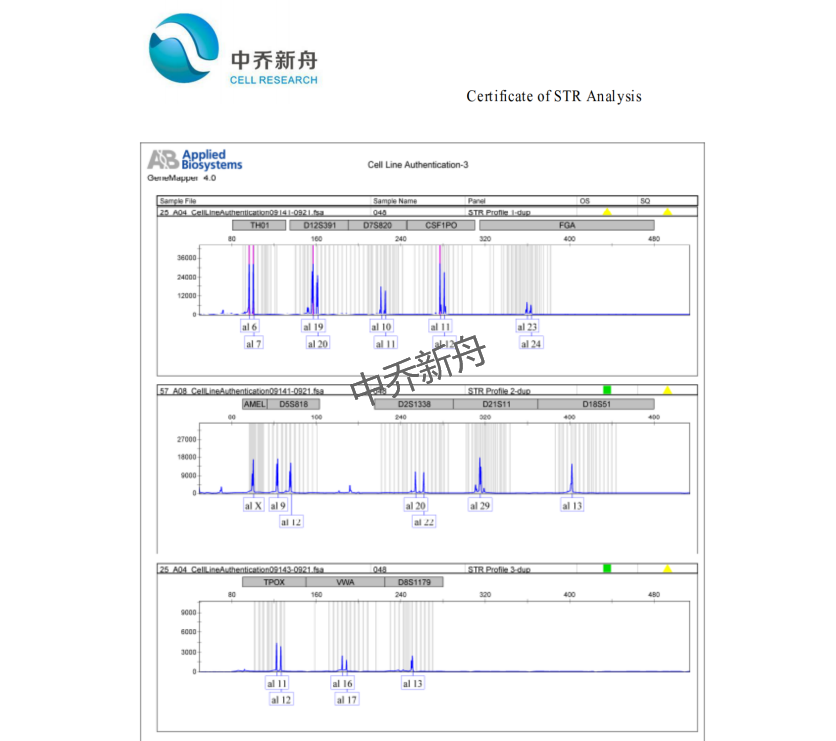


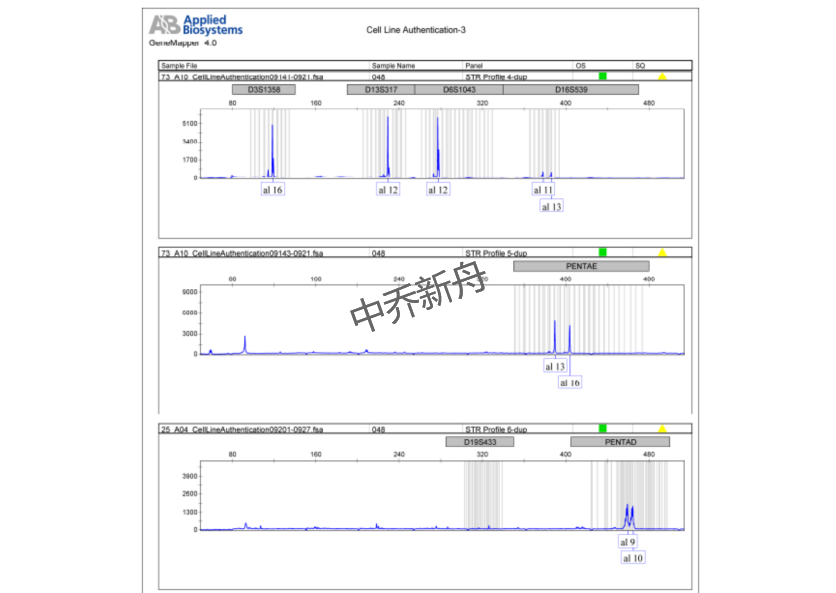


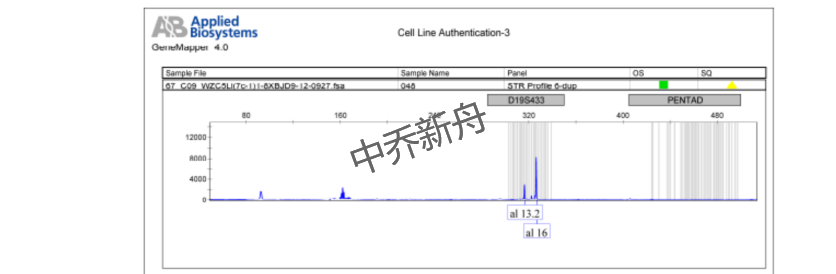


**Supplementary Materials 3 STR Profiling of the human mononuclear cell line THP-1**


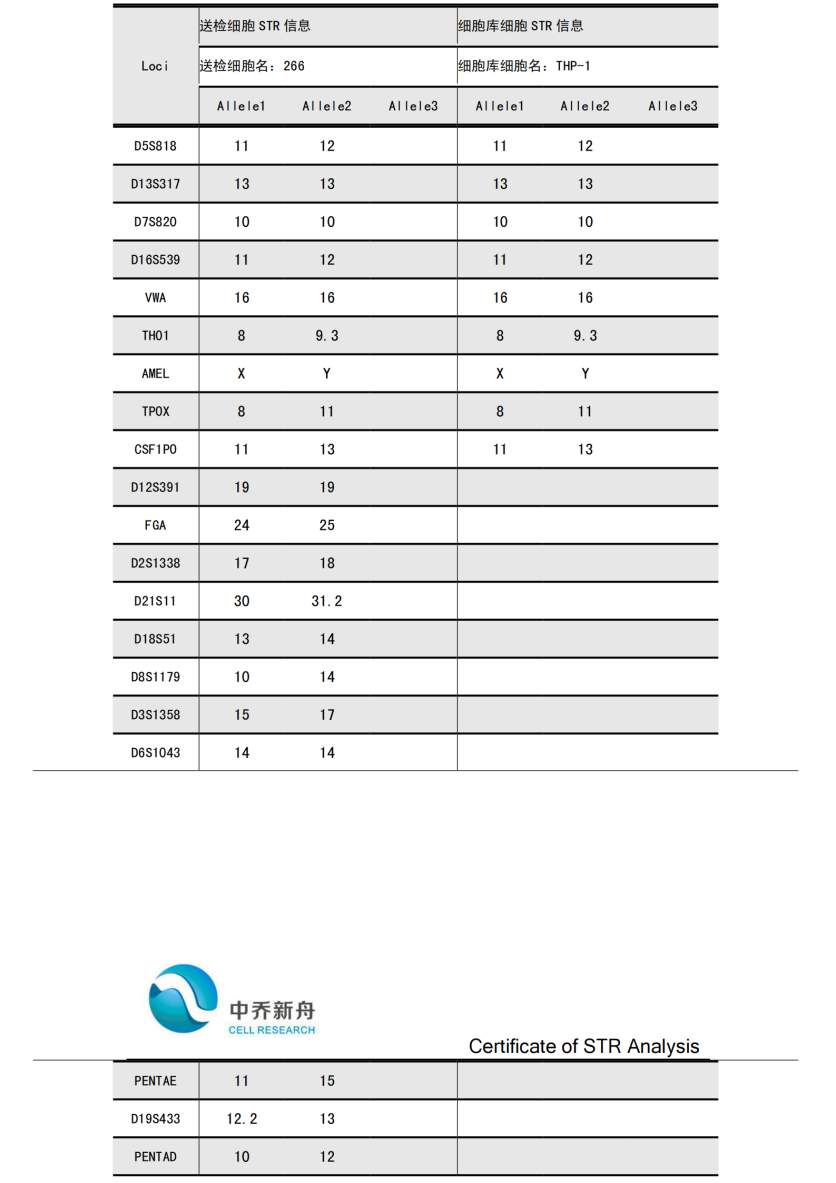


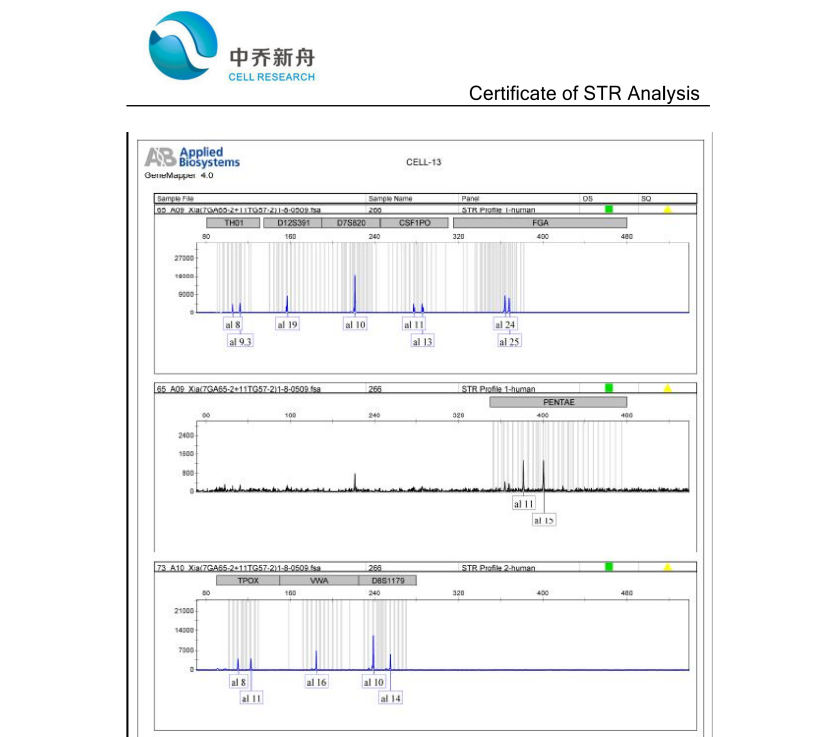


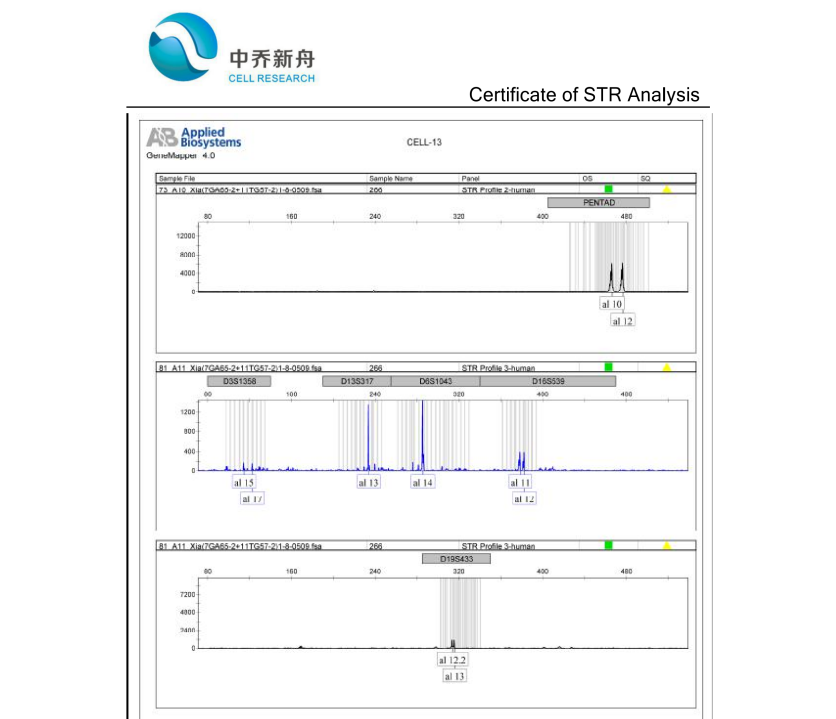


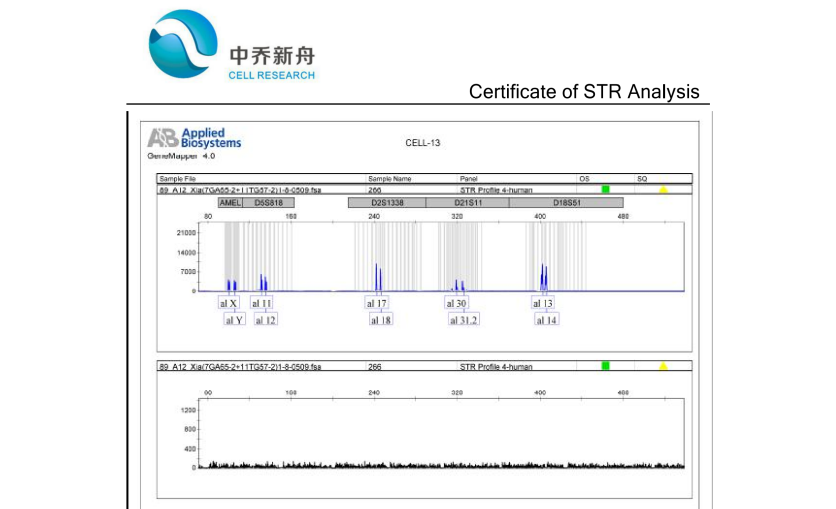

Supplement: Supplementary file 2 [file Table2.docx]
