# Supplementary figures and images for: Dextran sulfate inhibits the invasion, migration, and programmed death-ligand 1 expression in human gastric cancer cells by affecting the M2 tumor-associated macrophage polarization
Source: Front Oncol. 2025 Oct 10;15:1689053. doi: 10.3389/fonc.2025.1689053 (PMC12549276; doi:10.3389/fonc.2025.1689053)

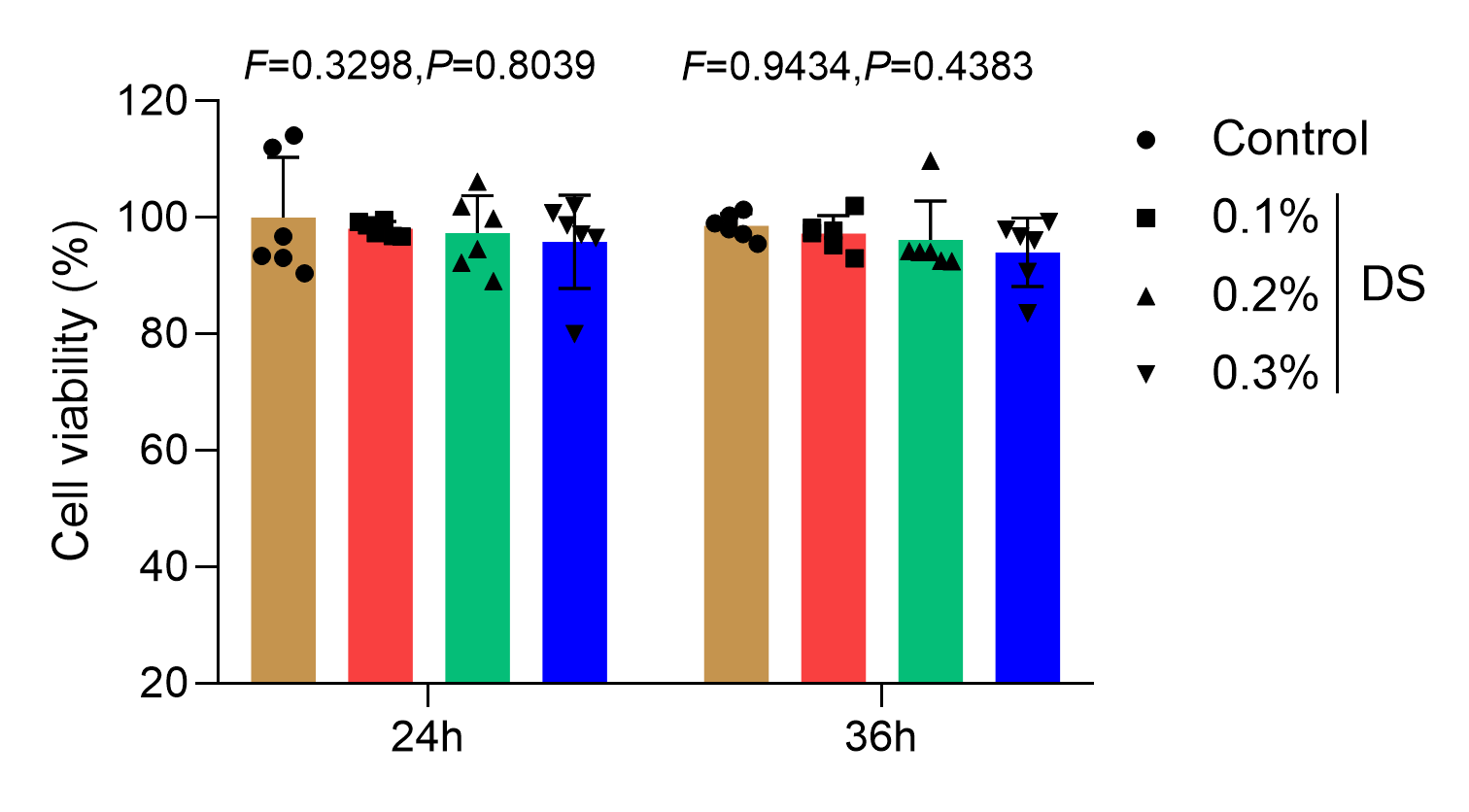

Supplement: Supplementary Figure 1 — | Effect of various concentrations of DS on the viability of GES-1 cells. Data were presented as mean ± S.E.M. and exact P value were shown in figure panels. [file Image1.tif]
